# Immunogenicity and Reactogenicity in Q Fever Vaccine Development

**DOI:** 10.3389/fimmu.2022.886810

**Published:** 2022-05-26

**Authors:** Alycia P. Fratzke, Erin J. van Schaik, James E. Samuel

**Affiliations:** ^1^Department of Veterinary Pathobiology, College of Veterinary Medicine and Biomedical Sciences, Texas A&M University, College Station, TX, United States; ^2^Department of Microbial Pathogenesis and Immunology, College of Medicine, Texas A&M University, Bryan, TX, United States

**Keywords:** Q fever, Coxiella burnetii, vaccine, reactogenicity, hypersensitivity, immunogenicity

## Abstract

*Coxiella burnetii* is an obligate intracellular bacterium which, in humans, causes the disease Q fever. Although Q fever is most often a mild, self-limiting respiratory disease, it can cause a range of severe syndromes including hepatitis, myocarditis, spontaneous abortion, chronic valvular endocarditis, and Q fever fatigue syndrome. This agent is endemic worldwide, except for New Zealand and Antarctica, transmitted *via* aerosols, persists in the environment for long periods, and is maintained through persistent infections in domestic livestock. Because of this, elimination of this bacterium is extremely challenging and vaccination is considered the best strategy for prevention of infection in humans. Many vaccines against *C. burnetii* have been developed, however, only a formalin-inactivated, whole cell vaccine derived from virulent *C. burnetii* is currently licensed for use in humans. Unfortunately, widespread use of this whole cell vaccine is impaired due to the severity of reactogenic responses associated with it. This reactogenicity continues to be a major barrier to access to preventative vaccines against *C. burnetii* and the pathogenesis of this remains only partially understood. This review provides an overview of past and current research on *C. burnetii* vaccines, our knowledge of immunogenicity and reactogenicity in *C. burnetii* vaccines, and future strategies to improve the safety of vaccines against *C. burnetii*.

## Introduction

Q fever is caused by *Coxiella burnetii*, an obligate, intracellular bacterium found worldwide except for New Zealand and Antarctica (1–3). In humans, Q fever mostly causes self-limiting respiratory illness, headache, and fever with as much as 60% of infected individuals being asymptomatic. However, in rare cases, Q fever causes severe acute syndromes such as hepatitis, myocarditis, placentitis and spontaneous abortion in pregnant individuals as well as chronic debilitating diseases: valvular endocarditis and Q fever fatigue syndrome (4–8). Valvular endocarditis caused by *C. burnetii* usually occurs in people with prior valvular defects and has a high risk of mortality in the absence of treatment (9, 10). Q fever fatigue syndrome causes chronic joint pain, lethargy and malaise which may last as much as ten years post-infection (2, 11).

*C. burnetii* is zoonotic and maintained through persistent infections in domestic ruminants (sheep, goat, cattle) and camelids. In regions where large numbers of these animals are produced or where small family farms are common, seroprevalence for anti-*C. burnetii* antibody titers can range from 19-45% (12–15). *C. burnetii* is predominantly transmitted to humans *via* aerosols, is highly resistant to heat and chemical disinfectants, and persists within contaminated environments for long periods. Because of this, the United States’ Centers for Disease Control and Prevention have designated *C. burnetii* as a category B select agent due to concerns for its potential use as a bioterrorism weapon (1). As such, development of a protective vaccine against *C. burnetii* is of high importance not only for occupationally at-risk populations but also for the safety of military personnel.

Vaccination is highly successful for prevention of numerous infectious diseases and vaccines against *C. burnetii* have been tested for use in humans since the discovery of this pathogen (16). However, despite decades of work, protective and safe vaccines against *C. burnetii* are not currently widely available and the single vaccine which is available is heavily restricted in its use. The only currently licensed vaccine for use in humans is Q-VAX (Seqirus), a whole cell, formalin-inactivated vaccine. Although this vaccine provides a high degree of long-term protection against infection, severe local and systemic reactions are frequently reported, especially in persons with prior exposure to *C. burnetii* (17, 18). Individuals receiving Q-VAX must first undergo pre-vaccination screening including anti-*C. burnetii* antibody titers and an intradermal skin test, a process which adds both cost and time to vaccination (19).

As result of this barrier to vaccine availability, many research groups have investigated novel vaccines against *C. burnetii* with varying degrees of success in equaling the protective efficacy of Q-VAX while reducing reactogenicity (**Table 1**). However, a growing wealth of information of the protective mechanisms of host immune responses to *C. burnetii*, investigations into the pathogenesis of reactogenicity to whole cell *C. burnetii* vaccines, and developments in novel vaccine technologies can help facilitate development of safer vaccines against this pathogen. This review provides an overview of past and current research on *C. burnetii* vaccines, our knowledge of immunogenicity and reactogenicity in *C. burnetii* vaccines, and future strategies to improve the safety of vaccines against *C. burnetii*.

**Table 1:** Summary table of published *C. burnetii* vaccine strategies tested in humans or animal models.

Vaccines	Efficacy	Reactogenicity	References
**Killed Whole Cell**			
Phase I Whole Cell (WCVI)	Near 100% protection over 5 years in humans	Severe persistent granulomas, systemic signs	(16) (20)
Phase II Whole Cell (WCVII)	Reduced protection compared to phase I in humans	No reduction in reactogenicity compared to WCVI	(21) (22)
* Δdot/icm*	Similar protection compared to WCVI in GPs	Reduced local erythema, significant local inflammation on histopathology	(22)
**Extract/Subunit**			
TCA	>90% protection from lethal challenge in mice and GPs	Local reactions which resolved after several days	(23) (24)
CMR	>95% protection in mice given 11*LD_50_	Local induration and erythema which resolved after a few days	(25) (26)
CMR – IT	Increased protection compared to SC route	No data	(27)
Sol II	Reduced weight loss and bacterial tissue burden in mice and GPs, prevented hypoxemia in NHPs	Reduced local inflammation observed on histopathology compared to WCVI	(28)
LPSI	Reduced mortality in mice, but only mild reduction in bacterial tissue burden	No data	(29) (30)
TLR Triagonists	In mice and GPs, variable but significant protection depending on adjuvant formulation	One adjuvant formulation reduced severity of reactions compared to WCVI	(31) (32)
m1E41920-KLH	In mice, reduced splenic bacterial burdens but less effective than LPSI	No data	(33)
**Live Attenuated**			
M-44	Antibody titers developed in 80% of humans inoculated	Fever for <24hrs, local reactions lasting 1-2 days, hepatitis, myocarditis, splenitis	(34) (35)
WCVII – IN	In mice, similar protection against pulmonary infection compared to WCVI	No data	(36)

Route of administration of vaccines is subcutaneous unless otherwise noted. IT, intratracheal; IN, intranasal; GP, guinea pig.

## Vaccines Against *C. burnetii*

*C. burnetii* was first discovered in 1935 in abattoir workers in Australia causing blood-culture negative infections characterized by pneumonia, fever, and severe headaches (37). Originally called abattoir fever, due to the uncertain cause of this disease and pressure to not associate this illness with local slaughterhouses, it was later renamed Query fever or Q fever (38). Edward Derrick, a pathologist in the Queensland Health Department, worked to collect samples and attempted to isolate the agent but was unable to do so and, believing that the agent must have been a type of virus, sent samples from infected animals to MacFarlane Burnet and Mavis Freeman who described an intracellular bacterium on histochemical stains of tissue from infected mice (38–40). Around this same time, Gordon Davis and Herald Cox were studying a new agent, called the Nine Mile fever virus, in the United States. It was later determined by Rolla Dyer, the at-the-time Chief of Infectious Diseases at the National Institutes of Health, that the agent of Q fever and Nine Mile fever were the same pathogen, which was ultimately named *Coxiella burnetii* (41, 42). A few years later, during World War II, several outbreaks of a severe febrile disease causing headache and pneumonia were described in Allied troops deployed to the Mediterranean (43, 44). This created an incentive for the United States to develop a preventative vaccine against *C. burnetii* for the protection of military personnel.

The first vaccines tested for *C. burnetii* were formalin-inactivated, whole cell vaccines derived from phase I bacteria (WCVI) originally isolated from an infected soldier of the 339^th^ Infantry during World War II (Henzerling strain) and Rolla Dyer, who developed a laboratory-acquired infection after visiting Herald Cox to study Nine Mile fever (Dyer strain) (44, 45). Evaluation of these vaccines in guinea pigs showed a mortality rate of 2% in vaccinated animals compared to 40-80% in unvaccinated animals after intraperitoneal injection. Additionally, both vaccines created from the Henzerling strain and Dyer strain showed significant cross-protection (16). An 18-month survey in slaughterhouse workers in Australia reported no cases of Q fever among 924 vaccinated individuals compared to 34 cases among 1349 unvaccinated workers (20). A second survey on the efficacy of WCVI showed nearly 100% protection against Q fever for a period of at least five years post-vaccination (46). Only two of the 2553 vaccinated individuals in the report developed Q fever, both of which occurred within the first two weeks after vaccination, suggesting that they had not developed full immunity from the vaccine at the time of exposure. Despite this high efficacy, a WCVI derived from the Henzerling strain is currently only licensed for use in humans in Australia as Q-VAX. This vaccine has not been approved for use in any other country due to the high rate of local and systemic reactions associated with it (17).

Adverse reactions to WCVI include local erythema, induration, pain and swelling as well as systemic signs including fever, headache, malaise, and joint pain (17, 23). These reactions usually occur starting one to two days post-vaccination and while systemic signs usually resolve within a few days, local induration is most severe at approximately two weeks after injection and may persistent for months (23, 47). Notably, reactions are more frequent and more severe in individuals with prior exposure to *C. burnetii* (23). Because of this, persons seeking vaccination with Q-VAX must undergo pre-vaccination screening including anti-*C. burnetii* antibody titers and intradermal skin testing. Despite this preventative testing, adverse responses are still common. A survey of titer and skin test-negative veterinary students in Australia reported local and systemic reactions in 98% and 60%, respectively. 30% of the reported local reactions were considered severe (erythema and swelling of >7.5 cm diameter at the injection site) (17).

To combat this reactogenicity, several other vaccines have been developed and tested over the decades since WCVI was created. These included killed phase II, live attenuated, chemical extracts, and subunit vaccines (21, 23, 25, 28, 32, 48). The most commonly used attenuated strains of *C. burnetii* involve phase variation which was first noted during early attempts to propagate the bacteria in embryonated yolk sacs. Multiple passages of *C. burnetii* in yolk sacs or cell culture lead to attenuation *via* the transition from expressing full-length phase I liposaccharide (LPS) to a truncated phase II LPS lacking the terminal O antigen (49). Phase I LPS is important in the pathogenesis of virulent *C. burnetii*. Phase I LPS blocks recognition of surface proteins by host toll-like receptor 2 (TLR2) and antagonizes TLR4, which normally recognizes bacterial LPS. In contrast, phase II *C. burnetii* has a marked reduction in virulence allowing it to be grown and studied in a biosafety level 2 facility, making it a potentially safer option for manufacturing an attenuated or killed vaccine (1). Unfortunately, early studies attempting to create a whole cell, killed phase II vaccine (WCVII) showed a marked reduction in protective efficacy compared to vaccines created from the virulent, phase I strains (21). Additionally, reactogenicity testing with WCVII showed no reduction in the severity of local reactive lesions compared to WCVI (22). A mutant strain of phase I *C. burnetii*, *Δdot/icm*, which lacks the type 4 secretion system (T4SS) was tested in guinea pigs as an alternate killed, whole cell vaccine. A single 25 µg dose of *Δdot/icm* protected guinea pigs against fever and splenomegaly during infection. In sensitized guinea pigs, *Δdot/icm* also produced less erythema at the injection site, but histopathologic evaluation of vaccine sites did not show a significant reduction in the severity of local inflammation compared to WCVI (22).

A live attenuated vaccine, M-44, was developed and used to vaccinate humans in Russia during the 1960’s. This vaccine was created by passaging the Grita strain of *C. burnetii* through embryonated yolk sacs for 44 generations resulting a marked reduction in virulence (48). In experiments in both animal models and humans, this vaccine showed significant protection against *C. burnetii* infection and reported a reduction in local and systemic reactions. Reactions in humans given a subcutaneous inoculation with M-44 were fever occurring for 24 hours at 2 to 3 days post-vaccination and erythema and swelling at the injection site at day 3 to 4, which resolved within 1 to 2 days (34). However, studies in vaccinated guinea pigs showed evidence of myocarditis, splenitis, and hepatitis, creating concerns about reversion of the attenuated vaccine to a virulent form, which precluded further evaluation in humans (35).

Some researchers used chemical extractions to try to separate the protective and reactogenic components of virulent *C. burnetii*. One group used trichloroacetic acid (TCA) extraction and another used the residue of chloroform:methanol (CMR) extraction. The TCA extract vaccine was composed of a complex of LPS, phospholipids, and proteins (50). Chloroform:methanol extraction of *C. burnetii* separates whole cell material into a soluble extract composed of lipids and a residue composed of LPS, proteins, and peptidoglycans (51). Both of these vaccines showed significant protection during challenge in animal models. The TCA vaccine showed >90% protection for mice and guinea pigs during lethal challenge (23). Similarly, a 1 µg dose of the CMR vaccine showed >95% protection in mice given an aerosol challenge of 11*LD_50_ (25). In humans, a prime-boost regimen with the CMR vaccine showed production of both anti-*C. burnetii* IgG titers and T cell priming (26). However, both of these vaccines still showed evidence of local and systemic reactions when injected in humans, though reports suggest that reactogenicity with these vaccines were somewhat less severe than those reported with WCVI (23, 26). Reactions were still more frequent in persons with positive titer or skin testing. Local reactions to the TCA vaccine ranged from transient erythema to local pain and swelling for several days with no reports of injection-site abscesses (23). In sensitized guinea pigs vaccinated with the CMR vaccine, although reactions were still frequent, local induration and erythema was less severe and resolved more quickly than those vaccinated with WCVI (52). Similarly, in humans receiving a prime-boost regimen of the CMR vaccine, 65% and 35% of individuals developed erythema and induration, respectively, after boost vaccination. However, reactions were most severe at 2 to 3 days post-injection and resolved within 7 days with no reports of systemic signs (26).

A recently published work described a solubilized antigen extract of phase II *C. burnetii*, Nine Mile strain (Sol II) using the detergents n-octylglucoside, anzergent 3-14, and sodium lauroyl sarcosinate. This extract was combined with the adjuvant CpG oligodendronucleotides (ODN), a TLR9 agonist, to produce an immunogenic vaccine. This vaccine provided similar protection against weight loss and bacterial tissue burden compared to the WCVI in guinea pigs and mice during challenge and, in rhesus macaques, Sol II protected against hypercapnia and hypoxemia during infection. Reactogenicity testing in sensitized guinea pigs showed significantly less erythema and induration than WCVI-elicited animals as well as a reduction in inflammation observed in vaccine sites on histopathology (28). TLR agonist adjuvants were also used in a subunit vaccine composed of six recombinant, immunodominant *C. burnetii* antigens. Different TLR agonist adjuvants, including TLR2, TLR4, TLR7, and TLR9 agonists, were conjugated to a triazine core to form triagonist adjuvants that were combined with the antigens to create several vaccine formulations. Evaluation in mice and guinea pigs showed variable levels of protection depending on the adjuvant formulation, with a greater level of protection with formulations that created a stronger Th1-type immune response. Reactogenicity testing in sensitized guinea pigs showed marked local reactions on histopathology of injection sites with some adjuvant formulations, while other adjuvant formulations showed a significant reduction in reactogenicity compared to WCVI. Interestingly, injection of unadjuvanted antigens in sensitized guinea pigs produce only minimal local inflammation, indicating that the adjuvant formulations were significantly contributing to reactogenicity of these vaccines (31, 32).

Phase I LPS (LPSI) of *C. burnetii* has been tested as a possible alternative vaccine. Antibodies targeting the phase I LPS have been shown to be a major contributor to the protective efficacy of WCVI as WCVII, which is antigenically similar to WCVI but lacks phase I LPS, is significantly less protective. In mice, the LPSI vaccine significantly reduced mortality during lethal challenge, but did not reduce bacterial burdens in mice infected intratracheally (29). A later study investigating the mechanisms of LPSI-mediated protection reported that both immune serum and splenocytes from LPSI-vaccinated mice protected naïve, wild-type mice during challenge, but in immunocompromised mice, only splenocytes and T cells provided significant protection (30). The authors in this study noted the possibility of contamination of the LPSI extract by protein antigens of *C. burnetii* as a potential explanation for the T cell-mediated immunity. A LPSI mimetic peptide, m1E41920, conjugated with keyhole limpet hemocyanin (KLH) was later tested as a potential novel vaccine, removing the possibility of protein contamination. A four dose regimen of m1E41920-KLH plus aluminum hydroxide resulted in significant reduction in splenic bacterial burden compared to naïve controls, but was less protective than the LPSI vaccine (33). Although specific studies of the reactogenicity of LPSI or m1E41920-KLH have not been published at the time of this review, the presence of severe reactogenicity in WCVII and other *C. burnetii* vaccines lacking LPSI suggest that this antigen may not significantly contribute to vaccine reactogenicity (22, 32).

Recently, some researchers have focused on alternate routes of immunization as a mechanism to enhance protective efficacy of *C. burnetii* vaccines and potentially reduce reactogenic responses. Intranasal inoculation of mice with live phase II *C. burnetii*, Nine Mile strain provided similar protection against challenge, as measured by splenomegaly and splenic bacterial burden, compared to vaccination with WCVI as well as significant cross-protection against infection with other *C. burnetii* strains (36). However, further testing is needed to investigate the safety profile of this live attenuated vaccine. A three-dose regimen of the CMR vaccine combined with CpG administered intratracheally showed greater protection against lung pathology and pulmonary bacterial burden than the same vaccine given subcutaneously (27). Although intratracheal inoculation may not be practical for human use, it provides promising evidence that mucosal vaccination may enhance the protective efficacy of *C. burnetii* vaccines. Further research is needed to determine whether mucosal administration of *C. burnetii* vaccines also reduces reactogenicity.

Lastly, of note are veterinary vaccines against *C. burnetii* to reduce both the impacts on infected herds and the potential spread of this pathogen to humans. Two vaccines against *C. burnetii* in ruminants exist for veterinary use: Coxevac (SEVA Santé Animale), a formalin-inactivated, whole cell, phase I vaccine, and Chlamyvax FQ (Merial), a combination vaccine containing phase II, formalin-inactivated, whole cell *C. burnetii* and inactivated *Chlamydia abortus* (also known as *Chlamydophila abortus*, formerly *Chlamydia psittaci* serotype 1) (53). However, difficulties in the use of *C. burnetii* vaccines in ruminants mirror those in humans. In pregnant goats, the phase II-derived Chlamyvax FQ did not provide significant protection against abortion or bacterial shedding. In contrast, surveys of ruminants vaccinated with the phase I-derived Coxevac showed a marked reduction in bacterial shedding and abortion rates compared to unvaccinated animals, but did not completely prevent bacterial shedding and, therefore, may not entirely prevent human exposure (54–56). Additionally, reactogenic responses to whole cell *C. burnetii* vaccines are also reported in ruminants including injection site induration and granulomas, fever, and decreased milk production (57, 58). Thus, development of more effective and less reactogenic vaccines against *C. burnetii* for use in animals as well as humans is warranted to both reduce animal morbidity and prevent human exposure and infection.

## Mechanisms of Protection Against *C. burnetii*

Understanding the mechanisms of protection against infectious agents is crucial for the development of effective vaccines. Several groups have published data investigating the infection and vaccine-mediated mechanisms of protective immunity against *C. burnetii.* Studies on post-infection immunity show that while both humoral and cellular responses play a role in control of infection, cell-mediated immunity is essential for bacterial clearance. A comparison of sera from acute and chronic Q fever patients showed that the humoral response is mainly driven by IgM during acute infection, while chronic infections showed stronger IgG and IgA responses (59). Mice deficient in B cells showed increased tissue pathology from infection and incubating *C. burnetii* with immune serum prior to inoculation into mice or guinea pigs reduced overall infectivity and the passive transfer of immune serum into naïve mice prior to infection resulted in more rapid clearance of bacterial (60–62). Similarly, *in vitro* studies showed that antibodies from immune serum enhanced dendritic cell maturation and potentiate phagocytosis and destruction by macrophages (63, 64). However, while passive transfer of immune serum inhibited *C. burnetii* infection in wild-type mice, immunocompromised mice still developed significant bacterial loads, indicating an essential role of T cells to control infection (30).

Several studies in mice have shown the importance of CD4 and CD8 T cells in controlling infection with *C. burnetii* (30, 62, 65). Mice depleted of both CD4 and CD8 T cells showed a marked increase in susceptibility to infection, while mice lacking either CD4 or CD8 T cells still controled and cleared bacteria (65). The pro-inflammatory cytokines IFNγ and TNFα, both produced by T cells, have also been shown to be essential for control of *C. burnetii* infection in mice (62). IFNγ activates the phagocytic activity and bacterial killing by macrophages while TNFα enhances immune cell recruitment and partially mediates IFNγ-dependent killing of engulfed bacteria by innate cells (62). Interestingly, mice depleted of CD4 T cells showed less pulmonary pathology than mice depleted of CD8 T cells suggesting that CD8 T cell-dependent immunity provides more significant protection (65). Supporting this, a separate study showed that while mice deficient in either major histocompatibility complex (MHC) I or MHCII develop more severe infections than wild-type mice, MHCI-deficient mice developed severe persistent infections with *C. burnetii*, where MHCII-deficient mice were still able to clear infection. This suggests that MHCI-restricted CD8 T cell responses play a critical role in bacterial control and clearance (66).

Investigations into the mechanisms of protective efficacy of WCVI similarly indicate vaccine-induced protection is mediated by both humoral and cellular components. Anti-phase I LPS antibodies are a major factor in why WCVII is less protective than WCVI, indicated by the protection provided by purified phase I LPS vaccines (30, 67). Although passive transfer of anti-*C. burnetii* IgM and IgG from WCVI-vaccinated mice inhibits infection, it does not prevent bacterial dissemination or clear infection in mice with T cell-deficiencies, supporting the essential role of T cells in both vaccine- and infection-generated immunity to *C. burnetii* (30, 68). In humans, antibody titers after vaccination did not correlate well with degree protection. While WCVI-vaccination resulted in a positive lymphocyte stimulation index in 85-95% of vaccinees, only 35-70% showed positive serum antibody titers (18, 69).

WCVI induces strong cell-mediated immune responses. Upon recognition by DCs, WCVI causes maturation and increased expression of CCR7. CCR7 enhances DC homing to draining lymph nodes and subsequently antigen presentation, increasing CD4 T cell responses (67). Vaccine-mediated protection with WCVI is markedly reduced in MHCII KO mice, indicating an important role for MHCII-dependent CD4 T cell responses. However, WCVI-vaccinated CD4 T cell KO mice still showed reduced bacterial tissue burdens compared to unvaccinated mice, suggesting MHCII mediates vaccine-induced protection through CD4 T cell-independent mechanisms, likely through the expansion of noncanonical T cell subsets in the absence of CD4 T cells (70). Tbet KO mice also showed a marked increase in splenomegaly, a common correlate of infection in animal models, when vaccinated with WCVI compared to WT mice while Stat6 KO and RORγT KO showed no loss in the protective efficacy of WCVI. Tbet regulates Th1 responses including production of IFNγ, activation of dendritic cells (DCs), and antibody production by B cells (70). Similar to Tbet KO mice, IFNγ KO mice vaccinated with WCVI showed significantly worse splenomegaly than did wild type mice. Tbet is also involved in antibody isotype switching and production of IgG2, which has been shown to play a significant role in the protective efficacy of WCVI (30, 70). As a result of these studies on the mechanisms of WCVI-induced protection, development of novel vaccines against *C. burnetii* should target the generation of similar Th1-skewed immune responses.

## Mechanisms of Reactogenicity With *C. burnetii* Vaccines

In contrast to studies on the mechanisms of protective efficacy of *C. burnetii* vaccines, far less work has been published on the mechanisms of WCVI-induced reactogenicity. Vaccines are designed to generate long-lasting immune memory by delivering antigens in conjunction with innate immune stimulation. Whole killed and attenuated live vaccines contain inherent immunostimulants, while protein and subunit vaccines often require the addition of adjuvants to initiate innate immune responses (71, 72). Once injected into the body, vaccine antigens and adjuvants activate cell surface receptors, such as toll-like receptors (TLRs), on immune cells and stromal cells. These surface receptors recognize evolutionarily conserved pathogen-associated molecular patterns (PAMPs) and damage-associated molecular patterns (DAMPs) and their binding leads to the production of proinflammatory cytokines. This initial local inflammation induces the recruitment and maturation of antigen-presenting cells (APCs), especially dendritic cells, which take up antigens and migrate to the local lymph nodes (73, 74). Mature dendritic cells present antigen and costimulatory signals to naïve T and B cells in germinal centers leading to activation and, hopefully, induction of long-term immune memory (75). Without the initial immune stimulation, dendritic cells fail to mature and, upon presenting antigen to T cells, lead to the formation of regulatory T cells (Tregs) and immunotolerance (76).

Vaccine reactogenicity is a result of the inflammatory responses incited by vaccine antigens and/or adjuvants. Clinically, this can present as a variety of symptoms which include both local and systemic responses. Local reactions include swelling, redness, pain, rash, and induration at the injection site, while systemic responses can present as fever, fatigue, myalgia, headache, and joint pain. Reactions may either be caused by innate or adaptive immune responses to vaccine antigens and/or adjuvants (74, 77). Symptoms caused by innate immune stimulation are most often self-limiting and generally considered tolerable given the benefits of vaccination. Adaptive-mediated reactions are often more severe and potentially can preclude vaccination. These reactions can be divided into acute type I responses, such as anaphylaxis, or delayed auto-immune disorders caused by type II, III, and IV responses, as discussed further in the following paragraphs (77).

Type I hypersensitivity responses are acute, often occurring within a few minutes to a few hours post-vaccination, and are mediated by preformed IgE on mast cells (77). These reactions are usually not in response to target antigens but rather arise against other vaccine components such as adjuvants or preservatives. Type I hypersensitivities can range from mild to severe and cause urticaria, angioedema, and anaphylaxis. Though serious, anaphylactic reactions are rare, on average, occurring <1 per one million doses across all vaccines (78). Type II hypersensitivity responses are mediated by antibodies, mainly IgM and IgG, targeting host proteins. Although rare, a type II hypersensitivity reaction has been proposed as the possible mechanism for vaccine-induced thrombotic thrombocytopenia associated with the adenovirus-vectored SARS-CoV-2 vaccine ChAdOx1 nCOV-19 (79). Type III hypersensitivity is caused by immune complex formation when antigen-specific IgG binds antigens and accumulates within local blood vessels. These immune complexes deposit in small capillaries and stimulate inflammation leading to localized pain, swelling, edema, and induration. Type III hypersensitivities have been reported with several types of vaccines including influenza, pneumococcal polysaccharide, tetanus-diphtheria-acellular pertussis (TDAP), measles-mumps-rubella (MMR), poliovirus, hepatitis A, meningococcal conjugate, and varicella vaccines (80). Type IV hypersensitivity is caused by memory T cell responses and may be mediated by CD4 and/or CD8 T cells. Vaccine-associated type IV hypersensitivities are usually Th1-mediated, while other type IV hypersensitivities, such as contact hypersensitivity, may be Th2-mediated. These can occur within a few hours of vaccination or as much as 2 to 3 weeks later. As such, association with vaccination can be tenuous and many different syndromes have been reported including maculopapular rash, Stevens-Johnson syndrome, toxic epidermal necrolysis, erythema multiforme, and Guillain-Barre syndrome among many others (81).

Type IV hypersensitivity is suspected to be the mechanism of reactogenicity for WCVI. Reactions to WCVI have a delayed onset, beginning at about 24 to 72 hours post-injection, and while systemic signs usually resolve within a few days, local swelling and induration may last for weeks and even years (16, 47, 82). In some instances, surgical excision was needed to resolve injection site granulomas (47). Adverse responses to WCVI are also more severe and more frequent in individuals who have had prior exposure to *C. burnetii*, but are also frequently reported in seronegative and skin test negative individuals (17). These reactions may be due to significant innate responses, however, some researchers have postulated that the intradermal skin test, which uses the same whole cell material as Q-VAX but at a lower dose, may be somewhat immunogenic (83). Histopathologic evaluation of local reactions to WCVI showed granulomatous and lymphocytic inflammation with abscesses and fibrosis (82). Unlike the tuberculin reaction, another type IV hypersensitivity response, the severe granulomatous inflammation reported with WCVI suggests a persistent antigen which is difficult to degrade or remove from the injection site (84). A recent publication characterized the local inflammation associated with WCVI in a sensitized mouse model. Local reactions displayed an influx of both CD4 and CD8 T cells and infiltrating CD4 T cells expressed IFNγ and IL17a, indicating a Th1-mediated hypersensitivity reaction. Similarly, CD4 T cells collected from the spleens of elicited mice showed an increase in IFNγ and IL17a expression, suggesting a possible mechanism for the systemic responses associated with WCVI hypersensitivity (85). However, further experiments are necessary to determine the roles of CD4 and CD8 T cells in mediating WCVI reactogenicity.

The underlying cause(s) of WCVI reactogenicity is currently unknown. Some studies postulated that the phase I LPS may be the main contributing antigen of WCVI reactogenicity, presumably because of its importance in mediating the protective efficacy of WCVI (23). However, recent studies have disproven this since WCVII is similarly reactogenic (22). Experiments evaluating the reactogenicity of a subunit vaccine paired with different adjuvant formulations indicated a significant role for innate immune stimulation in mediating WCVI reactogenicity. While the six *C. burnetii* antigens in the subunit vaccine were not reactogenic alone, the addition of adjuvants caused mild to severe reactogenicity depending on the adjuvant combination (32). This suggests that WCVI reactogenicity requires both innate and adaptive immune stimulation. Although WCVI does not contain any added adjuvants, whole cell vaccines contain innate immune stimulants such as LPS, surface lipoproteins, and microbial nucleic acids which are likely contributing to reactogenicity (73, 86, 87).

The presence of granulomatous inflammation with WCVI reactions also suggests a significant component of innate immune stimulation. Granulomatous inflammation with type IV hypersensitivities suggests an antigen which is difficult to degrade and it is possible that WCVI creates a depot effect which contributes to the chronicity of local reactions (84). *C. burnetii* has a polymorphic life cycle which includes large cell variants (LCVs), small cell variants (SCVs), and small dense bodies or endospores (88). LCVs are the metabolically active stage while SCVs and dense bodies are the inactive stages which are highly resistant to degradation and persistence in the environment (1, 88). *C. burnetii*’s ability to resist degradation in the environment could also translate to resistance to degradation in the host. In a study of blood and bone marrow samples from Q fever patients twelve years post-diagnosis, researchers isolated *C. burnetii* antigens from both recovered and chronic Q fever patients. While the samples were not infectious when injected into NOD/SCID mice, immunofluorescence detected *C. burnetii* antigens in the spleens of these mice and injection of samples into sensitized guinea pigs produced mild reactogenic responses similar to WCVI (7). Additionally, the use of formaldehyde to inactivate *C. burnetii*, a preservative which cross-links amino acid residues to prevent degradation, may also be contributing to persistence of WCVI antigens (89).

## Addressing Reactogenicity in Vaccine Development

Despite the difficulties of developing a safe and effective vaccine against *C. burnetii*, many new approaches to vaccine development provide potential pathways to resolve this issue. As vaccine strategies evolve, understanding the mechanisms behind protective efficacy and adverse responses to vaccination will expedite novel vaccine development. Several factors may play a role in vaccine reactogenicity and thus must be considered during development including antigen type, vaccine formulation, number and doses of immunizations, and route of administration (74, 90, 91).

Antigen type and composition may alter the rate of adverse responses to vaccination. Attenuated live vaccines often provide strong immunity by mimicking the kinetics of infection, but run the risk of prolonged infection or regression to virulent forms, especially in immunocompromised persons. Prolonged infections have been recorded with several live vaccines including those against poliovirus, measles, smallpox, varicella, yellow fever, and mycobacteriosis (81). While a low risk of vaccine-induced infection may be tolerated with highly prevalent and/or severe diseases, this risk becomes less accepted as prevalence decreases. The live oral polio vaccine is highly protective and provides greater mucosal immunity than the inactivated vaccine, but causes paralytic poliomyelitis at a rate of one in 500,000 doses, a rate considered unacceptable in regions with a low risk of infection (92). Although attenuated live vaccines against *C. burnetii* have shown promise in their protective efficacy, evidence that the attenuated M-44 vaccine induces multi-organ inflammation in animal models warrants a significant burden of proof for the safety of live Q fever vaccines, especially given this pathogen’s select agent status (35, 36, 48).

While killed vaccines have a higher overall safety profile compared to live vaccines, they still produce significant reactogenicity. Whole cell vaccines against *C. burnetii* cause severe chronic injection site reactions, but subunit vaccines show evidence of reduced reactogenicity depending on the vaccine formulation (28, 32, 47, 52). Several studies have identified immunodominant antigens of *C. burnetii* which may convey significant protection. Additionally, vaccines composed of defined antigens may use recombinant proteins to increase the safety of manufacturing processes by removing the need to culture virulent *C burnetii* (93–96). However, subunit vaccines often suffer from reduced overall immunogenicity as soluble antigens do not stimulate innate cell maturation leading to long-term memory. Thus, subunit vaccines often rely upon the addition of adjuvants or delivery systems which enhance immunogenicity but may also induce adverse responses.

For decades, aluminum salts were the main adjuvant in vaccines, but more recently several new adjuvants have been developed and are in use in human vaccines including virosomes, oil-in-water-emulsions such as MF59, TLR agonists such as monophosphoryl lipid A (MPLA) and CpG, and proprietary adjuvant mixtures such as AS01 (72, 87, 97, 98). While many of the mechanisms of adjuvants are still incompletely understood, recent studies have begun to elucidate these pathways and the resulting adaptive immune responses. This may allow vaccine developers to select adjuvants which enhance particular immune responses depending upon the agent of interest. Notable though is that certain combinations of adjuvants may result in immune responses not produced by the individual adjuvants alone as reported with AS01, a combination of MPLA and QS-21 (squalene oil-in-water emulsion) formulated in liposomes. MPLA activates TLR4 through TRIF-dependent signaling, while QS-21 activates caspase 1 in subcapsular sinus macrophages. The combination of these adjuvants in AS01 increases production of IFNγ by NK cells in the draining lymph node, enhancing Th1-type responses (72, 97). As such, adjuvant type, combination, and format may all alter responses to vaccination and further understanding of the mechanisms of adjuvants is needed to predict vaccine outcomes. Adjuvants have been shown to enhance local and sometimes systemic reactogenic responses compared to unadjuvanted controls, though in most cases these reactions are mild to moderate and resolve within a few days. However, adjuvants may also reduce the required amount of antigen and/or number of vaccine doses to achieve protective immunity and this dose-sparing may reduce the risk of hypersensitivity reactions with novel *C. burnetii* vaccines (91, 98).

Vaccine delivery systems are a promising area of vaccinology. Delivery systems in use or in development include liposomes, polymers, inorganic particles, viral and bacterial vectors, vesicles, emulsions, immune stimulating complexes (ISCOMs) and virus-like particles (VLPs). These delivery systems assemble antigens and adjuvants into particles which help traffic antigens to target tissues and mimic the size and distribution of endogenous adjuvants and antigens on pathogens (90, 91). Particulate vaccines ranging in size from 20 to 200 nm efficiently drain into lymphatics and local lymph nodes where follicular B cells and CD8+ DCs are present, enhancing both humoral and cell-mediated immune responses. Additionally, particulate vaccines with repetitive structures better induce B cell receptor cross-linking and subsequently B cell activation (90). Delivery systems may also reduce reactogenicity of adjuvants in vaccine formulations. Imidazoquinoline, a potent TLR7/8 agonist, is frequently used as a topical cancer therapeutic due to its ability to stimulate cell-mediated immune responses (86, 87). However, parenteral use of this adjuvant resulted in significant systemic effects including lymphopenia and renal and hepatic impairment. Lipidation of imidazoquinoline, as formulated in the adjuvant 3M-052, slowed dissemination from the injection site, reducing adverse systemic responses (99). Similarly, QS-21 is a potent enhancer of antibody and cell-mediated immunity but is associated with significant local reactogenicity. Incorporation of this adjuvant into ISCOMs or liposomes markedly reduced local reactions while maintaining its immunogenicity (97, 100). These studies are encouraging and indicate that vaccine delivery systems may be highly beneficial for both enhancing immunogenicity and reducing reactogenicity of novel vaccines.

Although most current vaccines are administered either *via* intramuscular or subcutaneous injection, alternative routes of delivery may help reduce reactogenicity while enhancing immune responses. Mucosal vaccines, especially intranasal and oral vaccines are particularly of interest due to enhancement of resident memory T cell (T_rm_) and secretory IgA responses (101–103). T_rm_ remain at the site of initial antigen exposure and can facilitate more rapid and tissue-specific responses to infection. However, these vaccines are often derived from live attenuated or whole-killed formulations due to the rapid degradation and clearance of subunit vaccines with mucosal administration. As a result, mucosal subunit and killed whole cell vaccines often require adjuvants to help stimulate immune responses. Bacterial enterotoxins are considered the most promising adjuvants for mucosal vaccination (101, 104). Mucosal vaccines containing derivatives of *E. coli* heat-labile toxin (LT) have been shown to enhance immune responses in preclinical and clinical testing (105–108). However, intranasal influenza vaccines adjuvanted with either wild-type LT or a detoxified derivative, LTK63, were associated with an increased risk of Bell’s palsy (109, 110). LThαK, another detoxified LT mutant, has shown a better safety profile and is currently being evaluated in clinical trials (111). Thus, while mucosal vaccines provide promise against *C. burnetii* and other pathogens, thorough safety evaluations are necessary to prevent adverse responses when combining adjuvants with alternative routes of vaccination.

Intradermal skin patches are another novel method of vaccine administration. These patches use microneedles to inject vaccine material into the dermis (112, 113). Although this method may enhance vaccine tolerance for individuals with an aversion to needle injection, the superficial distribution of antigen and adjuvant may result in more visible local reactions compared to subcutaneous and intramuscular injections (114, 115). Indeed, one of the reasons intramuscular injection may result in less injection-site pain is that skeletal muscle contains fewer pain receptors than skin (74). Thus, while alternative routes of vaccination provide new opportunities to address some of the problems immunogenicity and reactogenicity with injectable vaccines, new challenges for antigen delivery and induction of immune responses will need to be considered for their potential effects on the severity and types of adverse reactions observed with these novel strategies.

Lastly, some work has been done to investigate potential biomarkers of reactogenicity with the intent of predicting adverse responses early in vaccine development. Although work is still early in associating certain inflammatory cytokines and chemokines with local and systemic reactions, some surveys of individuals post-vaccination have identified some correlates of systemic adverse responses. Surveys of systemic cytokine levels have identified several potential biomarkers including PGE_2_, IL6, IFNγ, TNFα, MIP-1β, MCP-2, IP-10, CXCL10, CCL8, and CRP with systemic symptoms post-vaccination (74, 91, 116, 117). In the case of PGE_2_, a mechanism of action for its role in systemic reactogenic signs is known. PGE_2_ acts on E-type prostanoid (EP) receptors in the central nervous system causing many of the systemic symptoms associated with vaccine reactogenicity such as fever, headache, and fatigue (118). Alternative approaches including metabolomics, proteomics, and transcriptomics similarly provide an opportunity to investigate novel biomarkers to predict both safety and efficacy of vaccines and adjuvants (119, 120). These investigative approaches will become increasingly important as the wide array of antigen types, adjuvants, and delivery systems and their combinations makes testing every possible vaccine formulation empirically impractical. Thus, early predictors of vaccine efficacy and reactogenicity can significantly expedite vaccine development.

## Summary

Although our current understanding of the mechanisms of reactogenicity with Q fever vaccines is still incomplete, we are beginning to have some insights on how to address this issue. While whole cell vaccines provide significant protection against infection, they are associated with severe reactogenic responses and thus require costly pre-vaccination screening, preventing their widespread use. Reactogenicity with WCVI is associated with a Th1-type hypersensitivity response to vaccine antigen, but antibody-mediated immunity alone cannot control infection. Thus, T-cell mediated immune responses are necessary for a protective vaccine. In contrast, subunit vaccines which similarly produce Th1-skewed immune responses show promise in mitigating local reactions, though some show reduced efficacy compared to whole cell vaccines. A wide variety of adjuvants and vaccine delivery systems have yet to be tested for their use in a protective vaccine against *C. burnetii*, many of which can provide tailored immune responses to develop strong humoral and cellular immunity while reducing reactogenicity by trafficking antigens to targeted tissues and immune cells. Future studies should continue to investigate the mechanisms of both reactogenicity and immunogenicity of vaccines against *C. burnetii* so that development strategies for novel vaccines may be made deliberately rather than empirically. The availability of multiple animal models, the growing knowledge of the mechanisms of action of both old and new vaccine technologies, and the increasing use of bioinformatics to identify biomarkers of immunogenicity and reactogenicity can facilitate the development of novel vaccine candidates.

## Author Contributions

AF conceptualized, drafted, and edited the manuscript. EvS and JS edited and reviewed the manuscript. All authors contributed to the article and approved the submitted version.

## Funding

Support for this review was provided by the National Institutes of Health Institutional Training Grant T32 fellowship 5 OD 11083-11.

## Conflict of Interest

The authors declare that the research was conducted in the absence of any commercial or financial relationships that could be construed as a potential conflict of interest.

The handling editor declared a past co-authorship with the authors.

## Publisher’s Note

All claims expressed in this article are solely those of the authors and do not necessarily represent those of their affiliated organizations, or those of the publisher, the editors and the reviewers. Any product that may be evaluated in this article, or claim that may be made by its manufacturer, is not guaranteed or endorsed by the publisher.
